# Multivariate generalized multifactor dimensionality reduction to detect gene-gene interactions

**DOI:** 10.1186/1752-0509-7-S6-S15

**Published:** 2013-12-13

**Authors:** Jiin Choi, Taesung Park

**Affiliations:** 1 Department of Statistics, Seoul National University, Seoul, 151-747, South Korea

## Abstract

**Background:**

Recently, one of the greatest challenges in genome-wide association studies is to detect gene-gene and/or gene-environment interactions for common complex human diseases. Ritchie et al. (2001) proposed multifactor dimensionality reduction (MDR) method for interaction analysis. MDR is a combinatorial approach to reduce multi-locus genotypes into high-risk and low-risk groups. Although MDR has been widely used for case-control studies with binary phenotypes, several extensions have been proposed. One of these methods, a generalized MDR (GMDR) proposed by Lou et al. (2007), allows adjusting for covariates and applying to both dichotomous and continuous phenotypes. GMDR uses the residual score of a generalized linear model of phenotypes to assign either high-risk or low-risk group, while MDR uses the ratio of cases to controls.

**Methods:**

In this study, we propose multivariate GMDR, an extension of GMDR for multivariate phenotypes. Jointly analysing correlated multivariate phenotypes may have more power to detect susceptible genes and gene-gene interactions. We construct generalized estimating equations (GEE) with multivariate phenotypes to extend generalized linear models. Using the score vectors from GEE we discriminate high-risk from low-risk groups. We applied the multivariate GMDR method to the blood pressure data of the 7,546 subjects from the Korean Association Resource study: systolic blood pressure (SBP) and diastolic blood pressure (DBP). We compare the results of multivariate GMDR for SBP and DBP to the results from separate univariate GMDR for SBP and DBP, respectively. We also applied the multivariate GMDR method to the repeatedly measured hypertension status from 5,466 subjects and compared its result with those of univariate GMDR at each time point.

**Results:**

Results from the univariate GMDR and multivariate GMDR in two-locus model with both blood pressures and hypertension phenotypes indicate best combinations of SNPs whose interaction has significant association with risk for high blood pressures or hypertension. Although the test balanced accuracy (BA) of multivariate analysis was not always greater than that of univariate analysis, the multivariate BAs were more stable with smaller standard deviations.

**Conclusions:**

In this study, we have developed multivariate GMDR method using GEE approach. It is useful to use multivariate GMDR with correlated multiple phenotypes of interests.

## Background

Genome-wide association studies (GWAS) have been successfully conducted to detect disease susceptibility genes for common complex human diseases by focusing on associations between single-nucleotide polymorphisms (SNPs) and phenotypes [1]. While traditional methods for GWAS consider only one SNP at a time, some common complex human diseases such as diabetes, hypertension, and various types of cancers are known to be influenced by multiple genetic variants [2]. In addition, one of the greatest challenges in GWAS is to discover gene-gene and/or gene-environment interactions.

Classic logistic regression can be used to analyze the gene-gene interaction [3]. However, logistic regression suffers from an overfitting problem in high-order interactions [4]. Multifactor dimensionality reduction (MDR) method is a nonparametric, model-free, and combinatorial approach for interaction analysis by identification of a multi-locus model for association in case-control studies [5-9]. MDR method reduces multi-locus genotypes into two disease risk groups: high-risk and low-risk groups. If the ratio of cases and controls in a combination of genotypes is larger than a pre-assigned threshold *T *(e.g., *T *= 1), the cell of combination is labelled as "high risk", otherwise, "low risk". MDR method shows greater power for testing high-order interactions compared with logistic regression analysis [10]. Several statistical methods have been extended from MDR approach [11-16]. One of the extended methods of MDR is a generalized MDR (GMDR) proposed by Lou et al. [16]. GMDR method allows adjusting for covariates and applying to both dichotomous and continuous phenotypes; it uses the score-based statistic obtained from generalized linear model of phenotypes on the predictor-variable and covariates instead of the ratio of cases and controls in original MDR method.

These GWAS methods are generally implemented in a univariate framework analysing one phenotype at a time even though multiple phenotypes of interest are collected from a study population. In particular, pleiotropy that occurs due to potential genetic correlation between multiple phenotypic traits plays a role in pathogenesis of correlated human diseases [17]. Jointly analysing correlated multivariate phenotypes may have more power to detect susceptible genes and gene-gene interactions by using more information from data. Classic multivariate methods such as likelihood based mixed effects model [18,19] and generalize estimating equations (GEE) [20], and extended versions of these methods [21,22] can be applied to multivariate phenotypes of GWAS.

In this study, we have proposed multivariate GMDR method by extending GMDR method for the multivariate phenotypes. We construct GEE model with multivariate phenotypes to extend generalized linear models. The GEE approach is exceptionally useful method for the analysis of longitudinal data, especially when the response variable is discrete [23]. Using the score vectors from GEE, we discriminate high-risk from low-risk groups. The proposed multivariate GMDR method can also handle the repeatedly measured phenotypes.

We apply the proposed multivariate GMDR method to the Korean Association Resource study on blood pressure: systolic blood pressure (SBP) and diastolic blood pressure (DBP). A number of authors have investigated the genome-wide association studies on blood pressure and hypertension for Korean population [24-26] and for others [27-30]. However, not much work has been done for gene-gene interaction analyses. We compare the results of multivariate GMDR for SBP and DBP to the results from original univariate GMDR for SBP and DBP, respectively. We also apply the multivariate GMDR method to the repeated measured hypertension phenotypes and compare its result with those from univariate GMDR at each time point.

## Methods

### Multivariate GMDR

We introduce the generalized estimating equation (GEE) regarding a multivariate version of generalized linear model (GLM) which is implemented in GMDR. Let $y_{i}={\left(y_{i1},\cdots \;,y_{it}\right)}^{T}$ be the t×1 vector of the phenotypes for subject *i *(i=1,⋯,n), with expectation $\text{E}\left(Y_{it}\right)=\mu_{it}$. For the multivariate phenotype vector, $y_{i}$, we assume an underlying generalized linear model which can be written as

$$\eta_{i}=g\left(\mu_{i}\right)=X_{i}\beta +Z_{i}\gamma ,$$

where g(⋅) denotes a known one-to-one link function that is allowed to change with the characteristics of the different types of phenotype $y_{i}$. $X_{i}$ and $Z_{i}$ represent design matrices of genotype values and known covariate values including the unit component, respectively, and  β and  γ are vectors of their corresponding parameters, respectively. We assume that $y_{it}$ has a probability distribution belonging to the exponential family of distributions formed as

$$f\left(y_{it};\theta_{it},\phi \right)=\text{exp}\left\{\left[y_{it}\theta_{it}-b\left(\theta_{it}\right)\right]/\phi +c\left(y_{it},\phi \right)\right\}.$$

The GEE estimators of $\delta =\left(\beta^{⊺},\gamma^{⊺}\right)$ for marginal models can be obtained from the solution of a set of following generalized estimating equations:

$$U\left(\delta \right)=\overset{n}{\underset{i=1}{\sum }}{\left(\frac{\partial \mu_{i}}{\partial \delta }\right)}^{⊺}V_{i}^{-1}\left\{y_{i}-\mu_{i}\left(\delta \right)\right\}=0,$$

where $\partial \mu_{i}/\partial \delta$ is a matrix of derivatives whose *h*th column is $\partial \mu_{i}/\partial \delta_{h}$. $V_{i}$ is constructed as $V_{i}=\phi B_{i}^{1/2}R\left(\alpha \right)B_{i}^{1/2},$ where $B_{i}=\text{diag}\left(b^{{}^{\prime \prime }}\left(\theta_{it}\right)\right)$ is a diagonal matrix with main diagonal elements of variance function, $b^{{}^{\prime \prime }}\left(\theta_{it}\right)$, and  R is a correlation matrix. $V_{i}$ and  R are "working" covariance and correlation to distinguish them from the true covariance and correlation among $Y_{i}$, respectively. When we use canonical link function, $\partial \theta_{i}/\partial \eta_{i}$ is the identity matrix. Let $C_{i}$ be the matrix of predictor values with $X_{i}$ and $Z_{i}$ for subject *i*. By the chain rule,

$$\frac{\partial \mu_{i}}{\partial \delta }=\frac{\partial \mu_{i}}{\partial \theta_{i}}\frac{\partial \theta_{i}}{\partial \eta_{i}}\frac{\partial \eta_{i}}{\partial \delta }=B_{i}I_{i}C_{i}.$$

Then the score equations for  δ are

$$U\left(\delta \right)=\overset{n}{\underset{i=1}{\sum }}C_{i}^{⊺}B_{i}V_{i}^{-1}\left\{y_{i}-\mu_{i}\left(\delta \right)\right\}.$$

The expression, $B_{i}V_{i}^{-1}\left\{y_{i}-\mu_{i}\left(\delta \right)\right\}$ can be written as a vector of the residual of each phenotype, $y_{it}$. Thus, the residual score vector for individual *i *is defined as:

$$S_{i}^{*}=\left(\begin{array}{c} \begin{array}{c} S_{i1}\\ S_{i2} \end{array}\\ \begin{array}{c} \sim \\ S_{it} \end{array} \end{array}\right)={\hat{B}}_{i}{\hat{V}}_{i}^{-1}\left\{y_{i}-{\hat{\mu }}_{i}\right\},$$

where ${\hat{\mu }}_{i}=g^{-1}\left(Z_{i}\hat{\gamma }\right)$ and $\hat{\gamma }$ is estimator obtained from estimating equations under the null hypothesis $H_{0}:\beta =0$. ${\hat{B}}_{i}$ and ${\hat{V}}_{i}$ are calculated using ${\hat{\mu }}_{i}$. Based on this residual score vector, each individual with phenotypes is discriminated between case and control status. From the residual score vector for individual, we propose the aggregation for elements of the score vector, $S_{i}=\overset{t}{\underset{j=1}{\sum }}S_{ij}$, and use that as a prediction score for each individual. If the sum of prediction scores over those individuals who have the corresponding genotype combination is greater than a threshold value, assign 'high-risk' to the cell corresponding to the genotype combination. Otherwise, assign 'low-risk' to the cell.

### Data

Our primary outcomes are two types of blood pressure, systolic blood pressure (SBP) and diastolic blood pressure (DBP), and hypertension diagnosis of the Korean Association REsource (KARE) Consortium. The measurements of blood pressure were dichotomized at 140 mmHg for SBP and 90 mmHg for DBP, and denoted by SBP_B _and DBP_B_, respectively. We defined the hypertensive case as HP = 1 if SBP ≥ 140 mmHg or DBP ≥ 90 mmHg, and HP = 0, otherwise. Several genome-wide association studies (GWAS) have been performed on blood pressure by treating blood pressure as a quantitative trait [24-29]. In this study, we treated blood pressure as a binary trait HP representing whether the hypertension status is yes or no. Among 8,842 KARE subjects, 1,291 subjects were removed in the analysis due to anti-hypertensive therapy and drug treatments that could influence blood pressure. Additionally, 5 subjects were excluded because of missingness in SBP and body mass index (BMI). Of the 7,546 subjects considered in the study, 4,080 (54%) subjects were from urban community Ansan and the others were from rural community Ansung. For the study, the average age is 48.4 years for Ansan and 55.0 years for Ansung. There are three times of bi-yearly measured hypertensive status from 2001 to 2006, denoted by HP_1_, HP_2_, and HP_3_. Among 7,546 subjects, 2,080 subjects did not follow up at time 2 or 3. Subject characteristics are summarized in Table 1. The genomic DNAs were genotyped using Affymetrix Genome-Wide Human SNP Array 5.0. The quality control procedures were adopted such as missing genotype frequency > 0.5% and minor allele frequency (MAF) ≤ 0.01 at least on area. Finally a total of 7,546 individuals and 344,596 SNPs were included in the analysis of dichotomized SBP_B _and DBP_B_, while a total of 5,466 individuals and 344,309 SNPs were included in the analysis of repeatedly measured hypertension status.

**Table 1:** Subject characteristics of the KARE.

Phenotype		N(=7,546)	%
Recruit area			
	Ansung	3,466	45.9
	Ansan	4,080	54.1
Gender			
	Male	3,743	49.6
	Female	3,803	50.4
Systolic blood pressure			
	≥ 140	701	9.3
	< 140	6,845	90.7
Diastolic blood pressure			
	≥ 90	693	9.2
	< 90	6,853	90.8
Age (years)		**Mean**	**SD**
	Overall	51.4	8.79
	Ansung	55.0	8.82
	Ansan	48.4	7.51
Body mass index (kg/m^2^)			
	Overall	24.4	3.08
Hypertensive cases		N*(=5,466)	**%**
(SBP ≥ 140 or	HP_1 _(Time 1)	716	13.1
DBP ≥ 90)	HP_2 _(Time 2)	706	12.9
	HP_3 _(Time 3)	698	12.8

Abbreviations: DBP, diastolic blood pressure; KARE, Korean Association Resource; SBP, systolic blood pressure.

## Results

### Preliminary analyses

To compare multivariate analysis with univariate analysis, we first separately fit a logistic regression model for each dichotomized blood pressure measurement SBP_B _and DBP_B _with covariate adjustment for recruitment area, age, sex, and BMI. The correlation between SBP_B _and DBP_B _is 0.48. The multivariate analysis with two binary phenotypes (SBP_B_, DBP_B_) was conducted using the GEE approach. For the repeatedly measured hypertension status HP_1_, HP_2_, and HP_3_, we fit logistic models for each HP_i _and fit the GEE model for three HPs simultaneously. The pairwise correlations range from 0.32 to 0.36. In the GEE model, we assumed two types of genetic effect: homogeneous genetic effect and heterogeneous genetic effect for multivariate phenotypes. However, when we compared the effect sizes and p-values of homogeneous model with those of heterogeneous model, there was no strong evidence for supporting the homogeneous genetic effect. So, we present the results of the GEE model with heterogeneous genetic effects for multivariate phenotypes in both of blood pressures and repeatedly measured hypertension status.

To perform gene-gene interaction analysis using GMDR analyses, we first selected SNPs with strong marginal effects in univariate models and among those, we select the ones with strong effects in multivariate models. For SBP_B _and DBP_B _analysis, we selected the top 50 SNPs for each SBP_B _and DBP_B_. From these 100 SNPs, we chose 35 SNPs using a p-value criterion (< 1 × 10^-4^) from the GEE model. In a similar manner, we chose 34 SNPs for HP_1_, HP_2_, and HP_3 _by selecting the top 50 SNPs for each HP_i _using the same p-value criterion from their GEE model.

### Univariate logistic and multivariate GEE analyses of SBP_B _and DBP_B_

**Figure 1:**
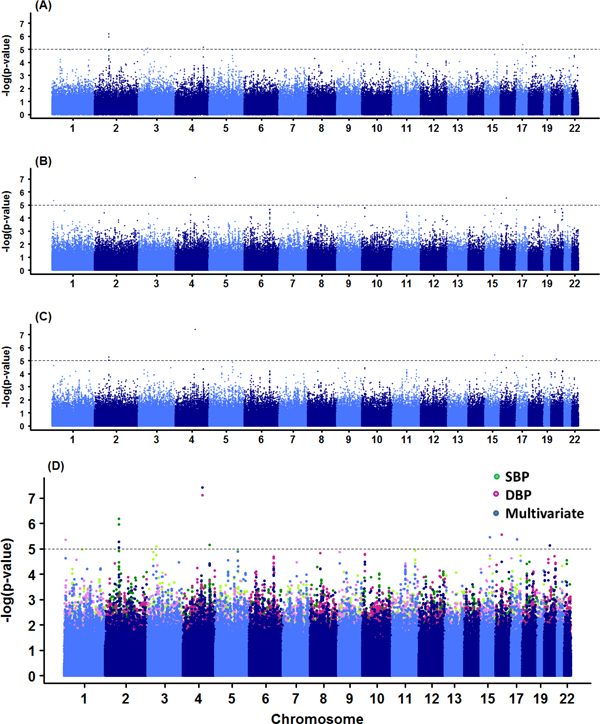
**Manhattan plots of SBP_B _and DBP_B _in univariate and multivariate analyses**. (A) SBP_B _in logistic regression (B) DBP_B _in logistic regression (C) Multivariate model with SBP_B _and DBP_B _(D) Overlay plot of (A)-(C).

We report results of GWA studies of dichotomized SBP_B _and DBP_B_, and their multivariate analyses. For SBP_B _and DBP_B_, the Manhattan plots are given in Figure 1. As summarized in Table 2, five SNPs for SBP_B _(rs1549022, rs2111464, rs12942470, rs2088983, and rs1768145) and three SNPs for DBP_B _(rs17045441, rs11866964, and rs7555790) were selected at the 10^-5 ^significance level. For multivariate GEE analysis for (SBP_B_, DBP_B_), six SNPs were selected: rs17045441, rs1378942, rs12942470, rs1549022, rs927833, and rs2111464. Among these six SNPs selected from multivariate GEE analysis, four SNPs were also found by univariate analysis but two SNPs (rs1378942 and rs2111464) were not. A gene *CSK *in which SNP rs1378942 is located has been reported as a hypertension susceptibility gene in the Korean population [25,26] and also in East Asians [30].

**Table 2:** Selected SNPs of SBP and DBP from univariate and multivariate analyses.

CHR	SNP	Gene symbol	SBP		DBP		Multivariate		
			
			Beta	P-value	Beta	P-value	Beta1	Beta2	P-value
1	rs7555790	*PEX14*	0.117	4.16E-03	**0.184**	**4.46E-06**	0.046	0.116	2.35E-05
2	rs2111464		**0.200**	**1.11E-06**	0.100	1.28E-02	**0.293**	**0.195**	**8.77E-06**
2	rs1549022		**0.207**	**6.52E-07**	0.111	5.89E-03	**0.295**	**0.202**	**5.23E-06**
3	rs1768145		**0.169**	**8.24E-06**	0.090	2.01E-02	0.233	0.161	7.95E-05
4	rs17045441	*ANK2*	0.065	1.06E-01	**0.199**	**7.69E-08**	**-0.090**	**0.058**	**3.91E-08**
4	rs2088983		**0.168**	**6.96E-06**	0.090	1.82E-02	0.234	0.162	4.54E-05
15	rs1378942	*CSK*	-0.189	2.50E-05	-0.192	1.85E-05	**-0.167**	**-0.182**	**3.49E-06**
16	rs11866964	*ZNF423*	-0.089	3.66E-02	**-0.206**	**2.78E-06**	0.036	-0.087	3.26E-05
17	rs12942470		**0.186**	**4.36E-06**	0.041	3.12E-01	**0.326**	**0.180**	**4.25E-06**
20	rs927833	*LOC100270679*	-0.127	2.31E-02	0.074	4.53E-02	**-0.343**	**-0.130**	**7.43E-06**

### Univariate logistic and multivariate GEE analyses of HP_1_, HP_2_, and HP_3_

**Figure 2:**
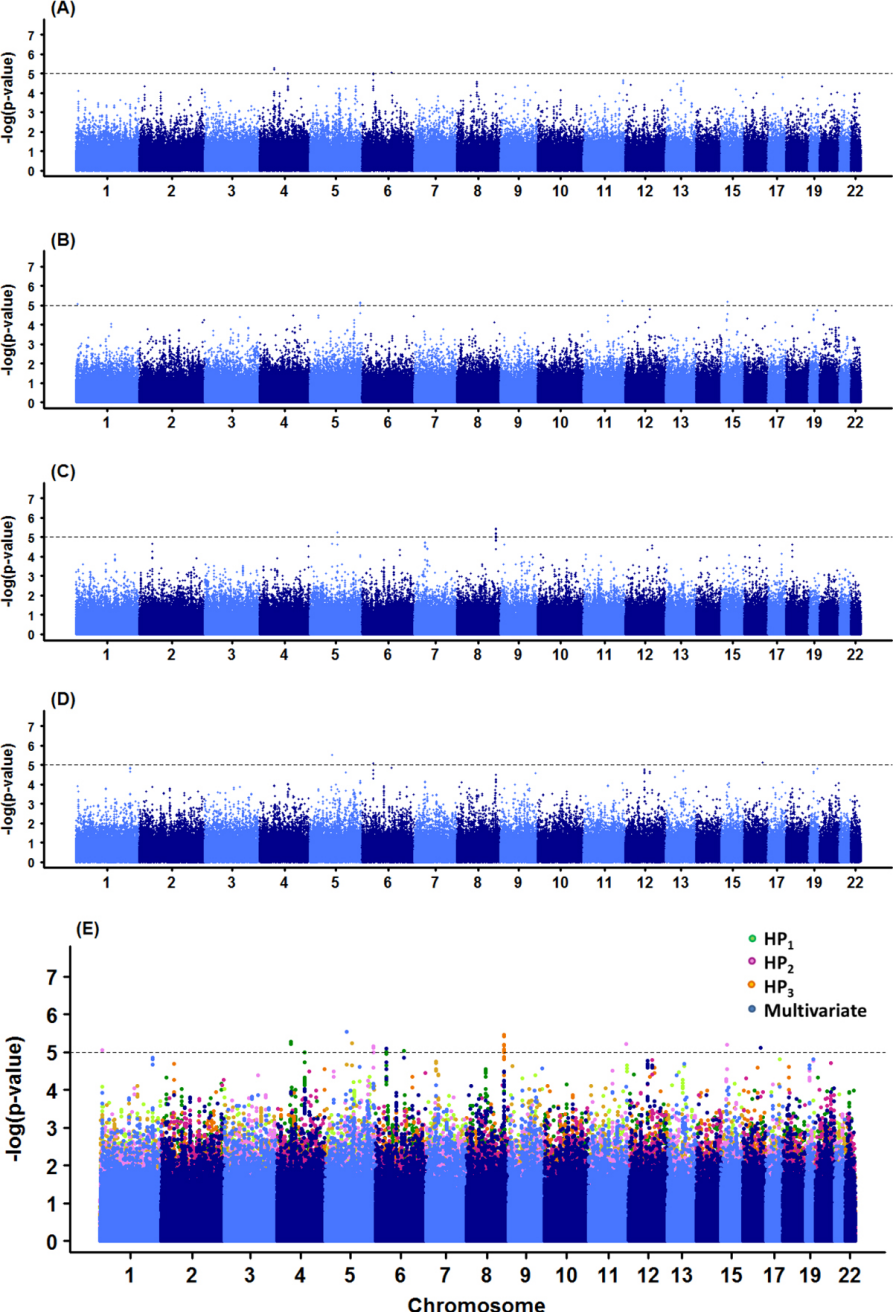
**Manhattan plots of longitudinal hypertension data in univariate and multivariate analyses**. (A) HP_1 _in logistic regression (B) HP_2 _in logistic regression (C) HP_3 _in logistic regression (D) Multivariate model with longitudinal hypertension (E) Overlay plot of (A)-(D).

We performed association analysis for the repeatedly measured binary hypertension phenotypes HP_1_, HP_2_, and HP_3_. First, the logistic regression model was fit for each HP_i _and multivariate analysis for (HP_1_, HP_2_, and HP_3_) was performed by GEE model. Manhattan plots are given in Figure 2. Nineteen SNPs were selected at 10^-5 ^significance level (Table 3): four for HP_1 _(rs17675997, rs2411259, rs4084097, and rs7751214), five for HP_2 _(rs4908736, rs17677051, rs4867707, rs550214, and rs11636344), and seven for HP_3 _(rs294082, rs4495407, rs10956596, rs6470947, rs4615555, rs4279577, and rs7465333), and three for multivariate HPs (rs12054837, rs4084097, and rs17722281). However, none of the identified SNPs were commonly observed by all three univariate analyses (Table 3). It might be due to the fact that the status of subject with hypertension is very volatile over time (Table 4) even though the proportion of hypertension risk was stable over time (Table 1). Thus the signals of association with hypertension were differently expressed over time. Among three SNPs from multivariate analysis, SNP rs4084097 was also associated with hypertension by univariate analysis at time 1. However, there were no common SNPs between multivariate GEE analysis and univariate analyses at times 2 and 3. One hypertension SNP at time 2, rs11636344, in *FBN1 *gene and another SNP rs17722281 of *WWOX *gene from multivariate have been previously found to be associated with hypertension in China population [31,32].

**Table 3:** Selected SNPs of longitudinal hypertension from univariate and multivariate analyses.

CHR	SNP	Gene symbol	HP_1_		HP_2_		HP_3_		Multivariate			
			
			Beta	P-value	Beta	P-value	Beta	P-value	Beta1	Beta2	Beta3	P-value
1	rs4908736		0.111	6.02E-03	0.178	**8.83E-06**	0.079	5.17E-02	0.110	0.178	0.079	1.21E-04
4	rs17675997		0.176	**6.16E-06**	0.051	2.11E-01	0.049	2.27E-01	0.175	0.051	0.048	1.27E-04
4	rs2411259	*LOC152578*	0.176	**5.33E-06**	0.051	2.06E-01	0.061	1.28E-01	0.174	0.051	0.061	1.13E-04
5	rs12054837	*ARSB*	-0.029	4.83E-01	-0.042	3.18E-01	0.162	2.12E-05	-0.031	-0.043	0.168	**2.95E-06**
5	rs294082		0.067	1.02E-01	0.087	3.28E-02	0.181	**5.80E-06**	0.068	0.087	0.181	1.71E-04
5	rs17677051		-0.086	3.79E-02	-0.188	**7.84E-06**	-0.089	3.16E-02	-0.081	-0.188	-0.093	8.09E-05
5	rs4867707		-0.088	3.22E-02	-0.189	**7.00E-06**	-0.091	2.77E-02	-0.083	-0.188	-0.095	6.80E-05
6	rs4084097		0.163	**9.61E-06**	-0.004	9.27E-01	0.092	1.68E-02	0.158	-0.005	0.095	**8.05E-06**
6	rs7751214	*EPHA7*	-0.191	**9.16E-06**	-0.009	8.26E-01	-0.099	1.85E-02	-0.190	-0.008	-0.100	1.39E-05
8	rs4495407		0.038	3.60E-01	0.012	7.74E-01	0.185	**8.40E-06**	0.036	0.012	0.189	5.59E-05
8	rs10956596		-0.044	2.82E-01	-0.047	2.58E-01	-0.185	**8.82E-06**	-0.043	-0.047	-0.188	1.23E-04
8	rs6470947		0.053	1.94E-01	0.023	5.69E-01	0.187	**6.69E-06**	0.053	0.023	0.190	6.05E-05
8	rs4615555		0.051	2.17E-01	0.030	4.69E-01	0.191	**3.81E-06**	0.049	0.029	0.194	3.34E-05
8	rs4279577		0.052	2.06E-01	0.031	4.56E-01	0.192	**3.44E-06**	0.051	0.030	0.196	3.26E-05
8	rs7465333		0.050	2.31E-01	0.031	4.57E-01	0.189	**6.33E-06**	0.048	0.031	0.193	5.75E-05
11	rs550214		0.081	4.38E-02	0.175	**6.09E-06**	0.102	1.01E-02	0.077	0.174	0.106	8.32E-05
15	rs11636344	*FBN1*	0.075	5.81E-02	0.167	**6.51E-06**	0.035	3.88E-01	0.073	0.166	0.037	1.06E-04
16	rs17722281	*WWOX*	-0.142	7.68E-04	-0.160	1.52E-04	0.034	4.16E-01	-0.140	-0.161	0.034	**7.66E-06**

**Table 4:** Transition of hypertensive case over time.

		**HP_1 _Time 1 (716)**			
		**Hypertension**		**Normal**	
		
		**HP_3 _Time 3 (288)**		**HP_3 _Time 3 (410)**	
		
		**Hypertension**	**Normal**	**Hypertension**	**Normal**
		
HP_2 _Time 2 (706)	Hyper- tension	166	154	147	239
	Normal	122	274	263	4101

Note that numbers within parentheses are the number of hypertensive case at each time point.

### Univariate GMDR and multivariate GMDR analyses of SBP_B _and DBP_B_

We present GMDR results to discover gene-gene and/or gene-environment interactions. For univariate GMDR analysis, logistic regression models for dichotomized SBP_B _and DBP_B _were constructed with area, age, sex, and BMI as covariates under the null hypothesis of no genetic effect. For multivariate GMDR analysis, the GEE model with same covariates was constructed. To reduce the computational burden, we focused on 35 SNPs selected from the preliminary analysis. All possible one and two locus models were fit for 35 SNPs. Through 10-fold-cross validation the best combination of loci with maximum train balanced accuracy (BA) which is average of sensitivity and specificity was chosen at each fold. To choose the final model, we considered cross-validation consistency (CVC) among a set of best combinations.

**Figure 3:**
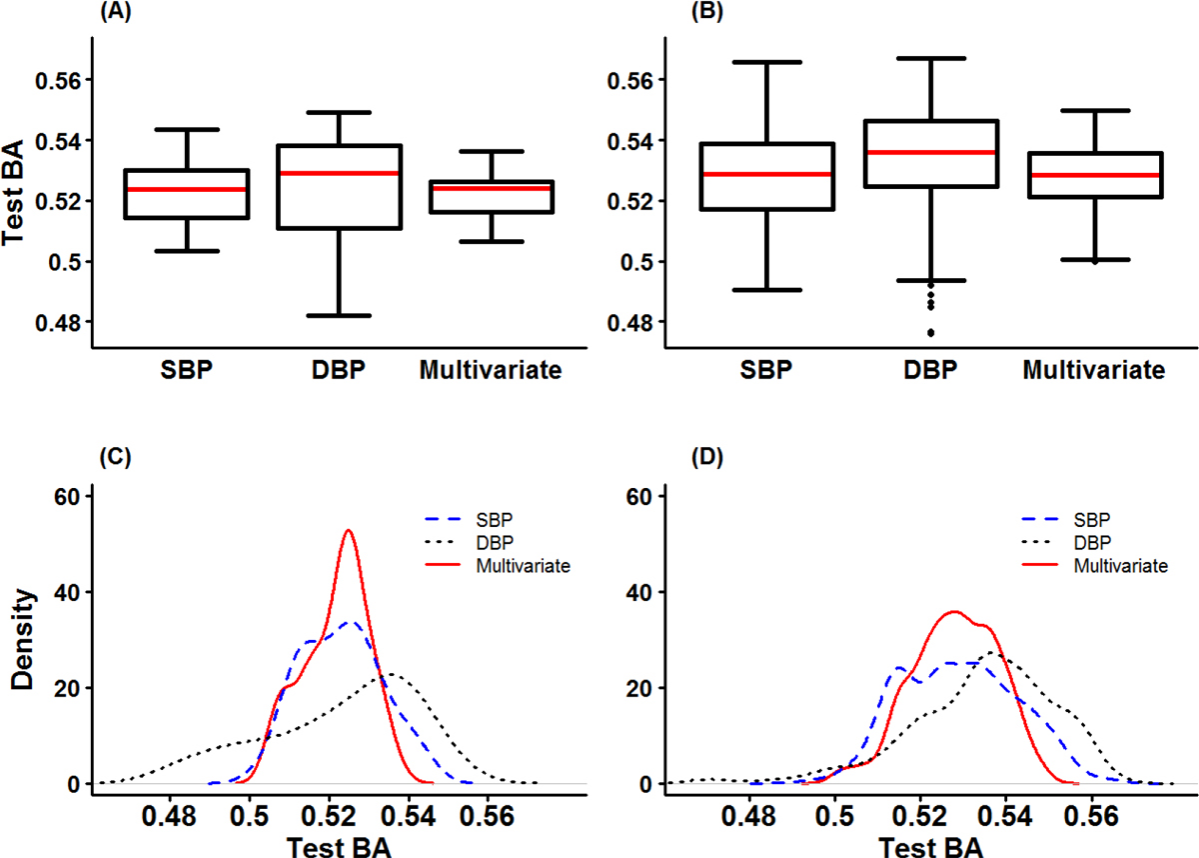
**Graphical descriptions for Test BA from GMDR analyses for SBP and DBP in univariate and multivariate analyses**. (A) Box plots of Test BA in one-locus model (B) Box plots of Test BA in two-locus model (C) Density plots of Test BA in one-locus model (D) Density plots of Test BA in two-locus model.

Table 5 summarizes the best model, Train BA, Test BA, and CVC from univariate GMDR and multivariate GMDR. For the purpose of comparison, we computed the p-values from the logistic models and GEE model for the SNPs in one-locus model of GMDR methods. The identified SNPs by GMDR methods also have significant p-values from these analyses: 6.51E-07 for SBP_B_, 4.21E-05 for DBP_B_, and 3.26E-05 for multivariate phenotypes. The best two-locus model of DBP_B _included one SNP, rs1378942, in *CSK *and another SNP, rs11866964, in *ZNF423 *implying that the interaction between *CSK *and *ZNF423 *genes was identified as a significant contributor to dichotomized DBP_B_. The test BAs of the one-locus models (two-locus model) for these SNPs were 0.545 and 0.549 (0.566) for rs1378942 and rs11866964, respectively. The best two-locus model from the multivariate GMDR included rs7555790 in *PEX14 *gene and rs11077135 in *A2BP1 *gene. The test BA of the one-locus models (two-locus model) for these SNPs were 0.526 and 0.532 (0.546), respectively. It seems that the contribution was from the joint effects of two genes rather than their main effects. The graphical descriptions for test BA are given in Figure 3. The median of test BA for multivariate GMDR is between median of SBP_B _and DBP_B _in both one and two-locus models. The distribution of test BA for multivariate GMDR is more concentrated than those of SBP_B _and DBP_B_.

**Table 5:** Comparison of results for SBP and DBP by GMDR and multivariate GMDR.

No. of Loci	Method	Best model	Train BA	Test BA	CVC
1	GMDR_SBP	rs1549022	0.544	0.544	6
	GMDR_DBP	rs11077135	0.548	0.547	7
	Multivariate GMDR	rs11866964	0.539	0.536	8
2	GMDR_SBP	rs2111464, rs12942470	0.566	0.566	7
	GMDR_DBP	rs1378942, rs11866964	0.566	0.566	3
	Multivariate GMDR	rs7555790, rs11077135	0.551	0.546	2

### Univariate GMDR and multivariate GMDR analyses of HP_1_, HP_2_, and HP_3_

**Figure 4:**
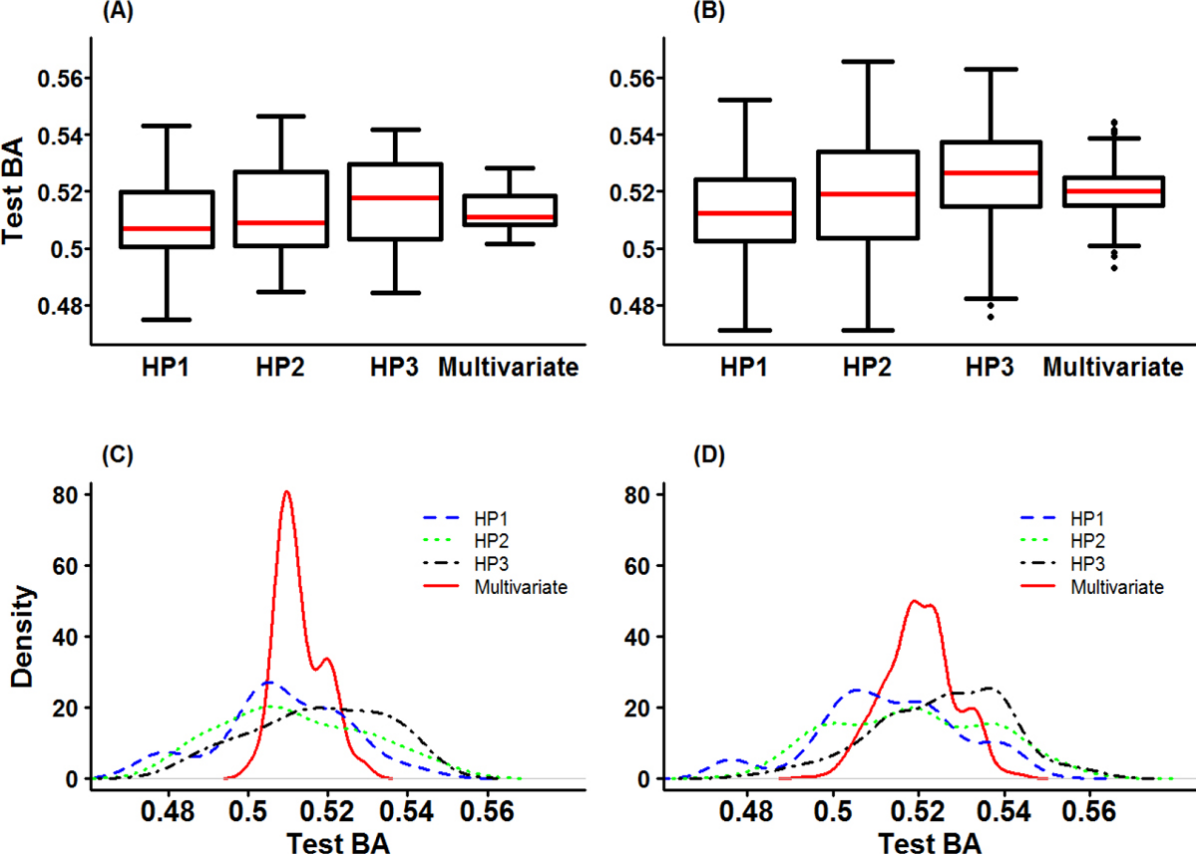
**Graphical descriptions for Test BA from GMDR analyses for longitudinal Hypertension in univariate and multivariate analyses**. (A) Box plots of Test BA in one-locus model (B) Box plots of Test BA in two-locus model (C) Density plots of Test BA in one-locus model (D) Density plots of Test BA in two-locus model.

The results of the univariate GMDR and multivariate GMDR are summarized in Table 6 for the repeatedly measured hypertension status HP_1_, HP_2_, and HP_3_. For these hypertension phenotypes, 34 SNPs selected from the preliminary analysis were included to GMDR mechanisms. All possible one and two locus models were fit for 34 SNPs. Not surprisingly, all different SNPs were identified in one-locus model. For the comparison between GMDR methods and classic method of logistic and GEE models, we report the p-values from the logistic models and GEE model for the identified SNPs from GMDR methods in one-locus models: 1.02E-05 for HP_1_, 1.59E-05 for HP_2_, 6.33E-06 for HP_3_, and 8.50E-05 for multivariate phenotypes. The identified SNPs by GMDR methods also have significant p-values from the classic methods. The best two-locus model from multivariate GMDR included rs7791839 in *CCDC129 *gene and rs7168365 in *WDR72 *implying that the interaction between *CCDC129 *and *WDR72 *genes was identified as a significant contributor to the repeatedly measured hypertension status. Box plots and density plots of test BA for GMDR and multivariate GMDR of HPs are given in Figure 4. Similar to the results of dichotomized SBP_B _and DBP_B_, the test BA for multivariate GMDR had a smaller deviation in the both one-and two-locus models.

**Table 6:** Comparison of results for longitudinal hypertension by GMDR and multivariate GMDR.

No. of Loci	Method	Best model	Train BA	Test BA	CVC
1	GMDR_ HP_1_	rs11097953	0.542	0.543	9
	GMDR_ HP_2_	rs11115097	0.545	0.546	5
	GMDR_ HP_3_	rs7465333	0.540	0.542	5
	Multivariate GMDR	rs7168365	0.529	0.528	9
2	GMDR_ HP_1_	rs11097953, rs7751214	0.555	0.540	6
	GMDR_ HP_2_	rs11115097, rs17722281	0.566	0.566	8
	GMDR_ HP_3_	rs7791839, rs6470947	0.563	0.563	9
	Multivariate GMDR	rs7791839, rs7168365	0.544	0.544	7

### Comparison of univariate GMDR and multivariate GMDR

**Figure 5:**
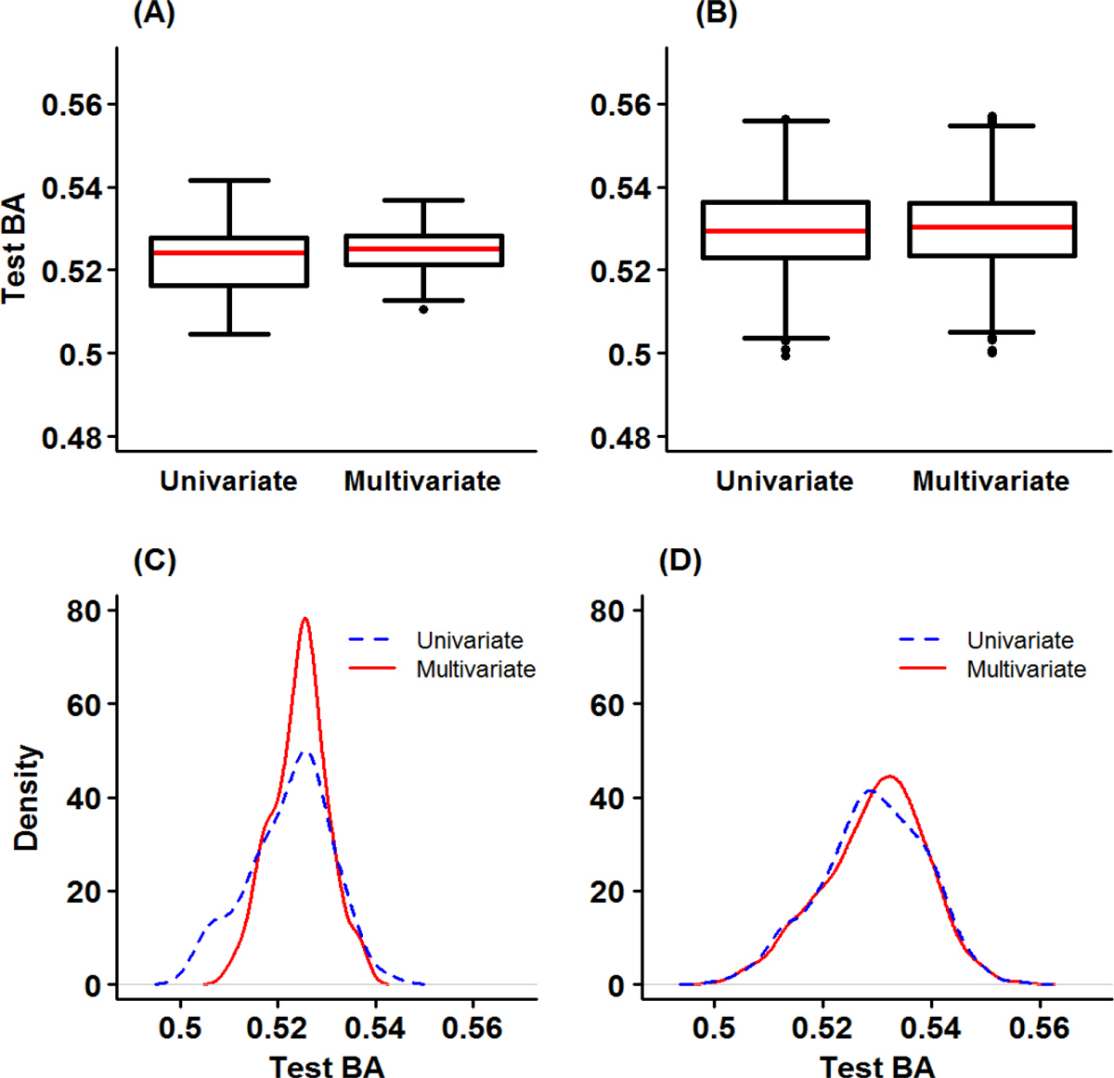
**Graphical descriptions for Test BA from univariate GMDR for hypertension at time 1(HP_1_) and multivariate GMDR for SBP and DBP**. (A) Box plots of Test BA in one-locus model (B) Box plots of Test BA in two-locus model (C) Density plots of Test BA in one-locus model (D) Density plots of Test BA in two-locus model.

We presented the results of univariate and multivariate GMDR by the same phenotypes in the previous two sub-sessions. However, those comparisons are not significantly meaningful to describe the usefulness of multivariate GMDR. Here, we compared the results from multivariate GMDR of SBP_B _and DBP_B _with the results from the GMDR of HP_1 _including the same individuals and candidate SNPs (Table 7). Because hypertension was defined by SBP_B _or DBP_B_, we can directly compare the performance of multivariate GMDR and univariate GMDR through those analyses. Multivariate GMDR and GMDR yielded the same best two-locus model. However, multivariate GMDR shows slightly better test BA than GMDR. Box plots of test BA for multivariate GMDR and GMDR from those two analyses are given in Figure 5. The test BA of multivariate model has smallest deviation also.

**Table 7:** Comparison of results for SBP and DBP by multivariate GMDR and hypertension at time 1 (HP_1_) by GMDR.

No. of Loci	Method	Best model	Train BA	Test BA	CVC
1	Multivariate GMDR with BPs	rs11866964	0.539	0.536	9
	GMDR with HP_1_	rs4811719	0.542	0.541	4
2	Multivariate GMDR with BPs	rs1338574, rs4811719	0.560	0.557	7
	GMDR with HP_1_	rs1338574, rs4811719	0.560	0.554	7

## Conclusions

In this paper, we have developed multivariate analysis for discovering gene-gene interaction, namely multivariate GMDR. Our multivariate GMDR analysis was developed by utilizing a GEE approach to multivariate phenotypes. Many studies emphasized the importance and the increase of power for multivariate analysis in GWAS [33-35]. Although MDR method has been developed in variety of manners [5-9], there have been no extensions to the multivariate analysis. We proposed multivariate GMDR analysis by utilizing the GEE model to calculate the prediction score to be a tool for reducing the multifactor dimensionality. The GEE approach is an extension of generalized linear models to the longitudinal data and handles both discrete and continuous phenotypes. Thus, our multivariate GMDR can be applicable to both discrete and continuous phenotypes.

Though real GWAS data analysis, we investigated the properties of multivariate GMDR. Firstly, the result of multivariate GMDR does not always coincide with that of GEE approach. That is, the best SNP set selected by multivariate GMDR does not always have the smallest p-value from GEE model. In our analysis, note that the SNP set selected by multivariate GMDR still tends to have quite a small p-value. Secondly, the test BAs of the multivariate GMDR is not always larger than those of univariate GMDR. As shown in Figures 3 to 5, the distribution of test BAs from the multivariate GMDR is different from those of univariate GMDR. The test BAs of multivariate GMDR are more densely distributed with a smaller standard deviation than those of univariate GMDR. Thus, a direct comparison of test BAs between multivariate GMDR and univariate GMDR may lead a misleading conclusion.

The proposed multivariate GMDR can be extended in many different ways. The modified version BAs which takes account for the distributional difference is expected to improve the performance of multivariate GMDR. The testing procedure using the modified BAs under the null distribution would enable us to demonstrate the increase of power of multivariate GMDR. A prediction score is defined as the sum of elements of the score vector from GEE model. We are currently working on several different weighting schemes for accounting various relationships between phenotypes. The weighted prediction score is also expected to improve the performance of multivariate GMDR. In the future studies, all these extensions will be evaluated through extensive simulation studies.

## Competing interests

The authors declare that they have no competing interests.

## Authors' contributions

JC and TP designed the study and JC carried out statistical analysis. TP coordinated the study. JC and TP wrote the manuscript. All authors read and approved the final manuscript.

## Declarations

The publication cost for this article was supported by the Seoul National University.

This article has been published as part of *BMC Systems Biology *Volume 7 Supplement 6, 2013: Selected articles from the 24th International Conference on Genome Informatics (GIW2013). The full contents of the supplement are available online at http://www.biomedcentral.com/bmcsystbiol/supplements/7/S6.
